# The Multipole Structure and Symmetry Classification of Even-Type Deviators Decomposed from the Material Tensor

**DOI:** 10.3390/ma14185388

**Published:** 2021-09-17

**Authors:** Changxin Tang, Wei Wan, Lei Zhang, Wennan Zou

**Affiliations:** 1 Institute of Photovoltaics, Nanchang University, Nanchang 330031, China; 5701118206@email.ncu.edu.cn; 2 School of Architecture Engineering and Planning, Jiujiang University, Jiujiang 332005, China; 6130085@jju.edu.cn; 3 Institute for Advanced Study, Nanchang University, Nanchang 330031, China; zouwn@ncu.edu.cn

**Keywords:** material tensor, Maxwell’s multipole representation, symmetry classification, deviator, multipole structure

## Abstract

The number of distinct components of a high-order material/physical tensor might be remarkably reduced if it has certain symmetry types due to the crystal structure of materials. An *n*th-order tensor could be decomposed into a direct sum of deviators where the order is not higher than *n,* then the symmetry classification of even-type deviators is the basis of the symmetry problem for arbitrary even-order physical tensors. Clearly, an *n*th-order deviator can be expressed as the traceless symmetric part of tensor product of *n* unit vectors multiplied by a positive scalar from Maxwell’s multipole representation. The set of these unit vectors shows the multipole structure of the deviator. Based on two steps of exclusion, the symmetry classifications of all even-type deviators are obtained by analyzing the geometric symmetry of the unit vector sets, and the general results are provided. Moreover, corresponding to each symmetry type of the even-type deviators up to sixth-order, the specific multipole structure of the unit vector set is given. This could help to identify the symmetry types of an unknown physical tensor and possible back-calculation of the involved physical coefficients.

## 1. Introduction

### 1.1. Nomenclature

Without additional indication, vectors and tensors are always supposed to be three dimensional and denoted by bold letters. The summation convention for repeated indices is implicit. The meanings of the main symbols used in this paper are shown in Table 1.

### 1.2. Material Symmetry and Physical Motivation

The physical properties of material exhibit orientation related anisotropy at a random point inside. However, the physical properties in some special orientations could be the same due to the symmetry of crystal structure at the micro-perspective. In the continuum mechanics, high-order tensors are used to describe the physical properties of materials, which are usually referred to as the physical tensors or constitutive tensors (for example, the elasticity tensor, the photo-elasticity tensor, the flexoelectric tensor and the sixth-order elasticity tensor involved in the theory of first strain gradient elasticity [1]). These higher-order tensors are difficult to handle, and the specific physical meanings of their components are not clear and simple. It is well known that the space expanded by these physical tensors can be divided into subspaces with equivalent symmetry classes. Two tensors in a same subspace are equivalences by sharing the same type of material symmetry, or through obtaining the symmetry point groups conjugate to each other. For example, the components of the stress and strain tensors are $\sigma_{ij}$ and $\varepsilon_{ij},$ respectively, in relation to the orthonormal basis. Hooke’s law takes the form below:

(1) $$\sigma_{ij}=C_{ijkl}\;\varepsilon_{kl},$$

where $C_{ijkl}$ represents the components of the fourth-order elasticity tensor ***C***. Here and after, the lower cases of Latin subscripts take the values of 1, 2 and 3. The material symmetry of an elastic body is exhibited in the collection of all orthogonal tensors, which are symmetry transformations of the tensor ***C***. The symmetry point group g(C) is defined as

(2) $$g\left(C\right)=\left\{Q\in O\left(3\right)|Q*C=C\right\}\;{\left(Q*C\right)}_{ijkl}=Q_{ip}Q_{iq}Q_{ir}Q_{is}C_{pqrs}.$$

Notably, Q represents the orthogonal tensor (Such as Qa·Qb=ab, for all pairs of vectors). The maximum group of the orthogonal tensors is O(3). For the subgroup of rotations (Orthogonal tensors with determinant equal to one), it is written as SO(3). The same type of material symmetry is obtained by two elasticity tensors if there is a conjugated relation stated as follows:

(3) $$g\left(C_{1}\right)\sim g\left(C_{2}\right)=\left\{Q\in \mathrm{SO}\left(3\right)|g\left(C_{1}\right)=Qg\left(C_{2}\right)Q^{T}\right\}.$$

where $Q^{T}$ represents the transposition of Q. Forte and Vianello’s paper [2] gave a more precise definition. There are seven non-isotropic symmetry types for the elasticity tensor ***C*** [2,3,4,5,6,7] and seven distinct systems for crystals.

The number of distinct components of a high-order physical tensor might be remarkably reduced if it has certain symmetry types. The matrix form of the elasticity tensor ***C*** was given in reference [3] for eight symmetry types. The number of distinct components varies from 2 (Isotropic materials) to 21 (Triclinic materials). Therefore, the symmetry classification of the tensors is useful and even becomes indispensable for the experimental identification or theoretical/numerical evaluation of the tensor components. In the tensor function theory, the determination of the number and types of tensor symmetry is also the basic problem for the construction of the tensor function and the determination of the clearest form of a tensor.

### 1.3. Deviator and Irreducible Decomposition

A tensor is called a deviator, and sometimes it is also called a harmonic tensor because it is traceless and symmetric about any pair of indices of its Cartesian tensor components. For example, a *n*th-order deviator, denoted by $H^{\left(n\right)}$ throughout this paper, with components satisfying

(4) $$H_{i_{1}i_{2}i_{3}\cdots i_{n}}=H_{i_{2}i_{1}i_{3}\cdots i_{n}}=H_{i_{3}i_{2}i_{1}\cdots i_{n}}=\cdots =H_{i_{n}i_{2}i_{3}\cdots i_{1}},H_{ssi_{3}\cdots i_{n}}=0.$$

Clearly, the scalars and vectors are zero-order and first-order deviators, respectively.

The general high-order tensor is quite complicated. For example, a *n*th-order general tensor ***T***^(*n*)^ contains 3*^n^* independent components. Therefore, it is impossible to obtain all symmetry types straightforwardly. In the theory of group representations, a *n*th-order tensor can be decomposed into a direct sum of deviators, the order of which is not higher than *n*. This is called irreducible or harmonic decomposition. Referring to the work of Zou et al. [8], the detailed direct sum is

(5) $$T^{\left(n\right)}=\sum_{j=1}^{j_{0}}\alpha_{j}⊕\sum_{j=1}^{j_{1}}v_{j}⊕\cdots ⊕\sum_{j=1}^{j_{s}}H_{j}^{\left(s\right)}⊕\cdots ⊕\sum_{j=1}^{j_{n}}H_{j}^{\left(n\right)}$$

where $j=1,\cdots ,j_{s}$ implies that the decomposition involves $j_{s}$ number of different *s*th-order deviators. In this paper, α and v represent scalar and vector, respectively, without any additional indication. The symbol ⊕ means that this formula is an abbreviated form. Actually, the items in formula (5) are *n*th-order irreducible tensors. For the complete form of the irreducible decomposition and the methods to manage such decomposition, please refer to reference [8]. As for the elasticity tensor ***C***, its irreducible decomposition takes the form of

(6) $$C_{ijkl}=\alpha^{1}\delta_{ij}\delta_{kl}+\alpha^{2}(\delta_{ik}\delta_{jl}+\delta_{il}\delta_{jk})+\left(\delta_{ij}H_{kl}^{1}+\delta_{kl}H_{ij}^{1}\right)\;+\left(\delta_{ik}H_{jl}^{2}+\delta_{jl}H_{ik}^{2}+\delta_{il}H_{jk}^{2}+\delta_{jk}H_{il}^{2}\right)+H_{ijkl}$$

or in brief,

(7) $$C=\sum_{j=1}^{2}\alpha_{j}⊕\sum_{j=1}^{2}H_{j}^{\left(2\right)}⊕H^{\left(4\right)},$$

where $\delta_{ij}$ is the Kronecker symbol, which is isotropic for any orthogonal tensor Q. This decomposition contains two independent scalars, two second-order and one fourth-order deviator, which was widely used in the symmetry problem of the elasticity tensor [2,4,5,6,7]. Meanwhile, the irreducible decomposition was used for the symmetry classification of the other physical tensors [9,10,11,12,13], and it was also applied in tensor analysis.

### 1.4. The Researches about Even-Order Physics Tensor

For the even-order general tensor ***T***^(2*n*)^, the irreducible decomposition yields that its symmetry group can be obtained from the intersection of the symmetry group of the relevant deviators:

(8) $$\begin{array}{l} g[T^{\left(2n\right)}]=\left\{g[\alpha_{1}]\cap \cdots \cap g[\alpha_{j_{0}}]\right\}\cap \left\{g[\epsilon v_{1}]\cap \cdots \cap g[\epsilon v_{j_{1}}]\right\}\cap \cdots \cap \left\{g[H_{1}^{\left(2\right)}]\cap \cdots \cap g[H_{j_{2}}^{\left(2\right)}]\right\}\\ \begin{array}{ccc} & & \cap \left\{g[\epsilon H_{1}^{\left(3\right)}]\cap \cdots \cap g[\epsilon H_{j_{3}}^{\left(3\right)}]\right\} \end{array}\cap \cdots \cap \left\{g[H_{1}^{\left(2n-1\right)}]\cap \cdots \cap g[H_{j_{2n-1}}^{\left(2n-1\right)}]\right\}\cap g[H^{\left(2n\right)}] \end{array}$$

In above, the combination ϵH could be ϵ⊗H or ϵ·H. As noted, only the odd-order deviators are combined with the permutation tensor ϵ. Then, ϵH will be an even-order tensor and its symmetry group contains $\bar{I}$ (central inversion). Analogously, in the irreducible decomposition of an odd-order general tensor, the even-order deviators are combined with the permutation tensor ϵ. Therefore, the *n*th-order deviators in the irreducible decompositions will be divided into two types: the even-type $H_{e}^{\left(n\right)}$ and the odd-type $H_{o}^{\left(n\right)}$, respectively. The definition of even-type deviator is listed as below:

(9) $$H_{e}^{\left(n\right)}\left\{ \begin{array}{l} H^{\left(n\right)}\;\mathrm{when}\;n\;\mathrm{is}\;\mathrm{even},\\ \epsilon H^{\left(n\right)}\;\mathrm{when}\;n\;\mathrm{is}\;\mathrm{odd}. \end{array}\right.$$

To solve the problem about symmetry classification of the even-order tensors, obviously, the two following tasks should be done. The first is to obtain the symmetry types of even-type deviators; the second is to find a solution to do the intersection of formula (8).

According to this idea, there are abundant results about the symmetry classification of high even-order tensors. Forte and Vianello [2] proved that the number of symmetry types of the elasticity tensor is eight for the first time. Their study greatly promoted a comprehensive understanding and resulted in a series of studies on the symmetry of elasticity tensor [3,4,5,6,7]. The relevant methods were also applied to the other fourth-order physical tensors like the photo-elasticity tensor [9] and the flexoelectric tensor [10].

The deviator is known as a relatively simple tensor of higher-order (the number of distinct components of a *n*th-order deviator is 2*n* + 1). However, the structure of the high order deviator is still complicated and it is hard to obtain its symmetry types. Since the $\bar{I}$ was contained in symmetry group of even-order tensors, the corresponding symmetry type is reduced to a subgroup of SO(3). A mature approach has been widely used for the symmetry problems of even-order deviator [2,3,4,5,6,9,10]. It is explained as follows: Firstly, the isomorphism relation between the spaces of *n*th-order deviators and the spaces of harmonic polynomials with degree *n* is established. Then, the space of harmonic polynomials is decomposed to terms that are invariant under the specific rotation. The rotation is known as the Cartan decomposition [14]. However, the lack of achievement of general results is regarded as an obvious disadvantage for this method.

### 1.5. The Symmetry Classification of Even-Type Deviators

Olive and Auffray [15] derived a general conclusion on the number of symmetry types of even-order tensors. They proposed a tool named clips operator, in order to execute the intersection of symmetry types of an even-type deviator couple. As for the symmetry types of arbitrary order even-type deviators, they directly referred to the results of Ihrig and Golubitsky [16]. The modern definition of symmetry classification of tensors was introduced by Huo and Del Piero [17] in 1991 and further modified by Forte and Vianello [2] in 1996. The latter one is now accepted and applied extensively. It is obvious that the results given by Ihrig and Golubitsky [16] in 1984 were earlier than the modern definition of symmetry classification, and a reexamination is required due to the potential shortcomings.

Unlike the existing methods described in previous articles, the symmetry of an arbitrary order even-type deviator is classified by an exclusion of two steps in this paper. The preliminary results are obtained based on the order of tensor, which is well known in existing papers [18]. Then, by utilizing Maxwell’s multipole representation [19], the deviator is expressed in terms of a scalar module and a unit vector set, which could be used to clarify the anisotropic structures and make accurate exclusion about the symmetry types of the deviator. Obviously, the set of unit vectors indicates the multipole structure of deviators, which is a nice geometric view of the deviator. The multipole structure has already been applied in the representation theory of the tensor function to find invariants [19]. The unit vector sets were also used to identify the symmetry type of the physical tensor, the components of which are related to an arbitrarily oriented coordinate system [7,12]. Thus, the specific unit vector sets corresponding to every symmetry type of even-type deviator would be given out, which is one of the contents in this paper.

As noted earlier, the symmetry classification of the even-type deviator is the basis for the symmetry problem of an arbitrary even-order physical tensor. The method of this paper can be extended to the odd-type deviators and the arbitrary order physical tensors easily. The rest of this paper is organized as below. In Section 2, the method route of symmetry classification is given and the symmetry types of even-type deviators are preliminarily determined based on the order of tensor. As the key of this paper, Maxwell’s multipole representation is introduced in this section too. In Section 3, by utilizing Maxwell’s multipole representation, the possible symmetry types of even-type deviators are finally determined. In the end, some refined conclusions and a brief discussion are given in Section 4.

## 2. Methods and Related Theory

### 2.1. The Method Route of Symmetry Classification

**Figure 1:**
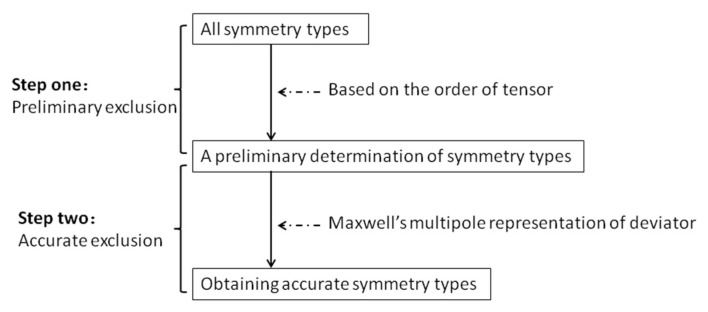
The method route of symmetry classification.

In this paper, the method of symmetry classification is according to the idea of exclusion. Two steps are shown in Figure 1. The first step is to get a preliminary determination of symmetry types based on the order of the tensor. This results are given by the reference [18] first. In Section 2.2, the preliminary results and corresponding derivation process are reorganized, such as the classification theorem, which collects all possible symmetry types of tensor and the methods to make a preliminary exclusion from the order of the tensor. The second step is to obtain accurate results. The key of this step is the application of Maxwell’s multipole representation. Hence, Section 2.3 is presented to introduce Maxwell’s multipole representation, which has been described in the paper [19]. Above all, the detailed process and the accurate results are introduced in Section 3 based on the contents of this section.

### 2.2. Preliminary Results

A fundamental result of symmetry classification is that the collection of involved groups may cover the whole O(3)-closed subgroups and modulo conjugation [18,20,21], which is to collect all possible symmetry types of tensor. The collection is known as the classification theorem. The article of Zheng and Boehler [18] described this theorem in detail. It states that:

*A three-dimensional point group is conjugate to one of the groups given in Table 2.*

According to the definition of the symmetry type of a tensor, there is a straightforward corollary that states that the symmetry types of each tensor are described by one of the point groups in Table 2. Additionally, a very important conclusion (which was reduced to Theorem 2 in the article of Bona et al. [5]) is proposed and proven. It is stated as below:

*If an nth-order tensor is an invariant under a (n + 1)-fold rotation (k ≥ 1) about a given axis, then it will be an invariant under any rotation about this axis.*

Although the possible symmetry types are numerous in Table 2, the range of scope could be reduced easily according to the tensor’s order. For even-type tensors, the following corollary was firstly reported by Zheng and Boehler [18], and it was rechecked by this research:

**Corollary** **1.** *For even-type deviator $H_{e}^{\left(n\right)}\left(n\geq 2\right)$, the symmetry group is conjugate to one of the following:*

(10) $$C_{i},\;C_{3i},\;C_{5i},\;\cdots ,\;C_{\left(2k-1\right)i},\;\mathrm{for}\;1\leq 2k-1\leq n;\;D_{3d},\;D_{5d},\;D_{7d},\;\cdots ,\;D_{\left(2k-1\right)d},\;\mathrm{for}\;3\leq 2k-1\leq n;\;C_{2h},\;C_{4h},\;C_{6h},\;\cdots ,\;C_{\left(2k\right)h},\;\mathrm{for}\;2\leq 2k\leq n;\;D_{2h},\;D_{4h},\;D_{6h},\;\cdots ,\;D_{\left(2k\right)h},\;\mathrm{for}\;2\leq 2k\leq n;\;C_{\infty h},\;D_{\infty h},\;T_{h}\left(n\geq 3\right),\;O_{h}\left(n\geq 4\right),\;I_{h}\left(n\geq 5\right).$$

Of course, Corollary 1 also works for the even-order general tensor ***T***^(2*n*)^.

Thus, the first step of a preliminary determination on symmetry types of even-type deviators is achieved. Although the symmetry types given by formula (10) should be further refined, there are only a few symmetry types that should be ruled out.

### 2.3. Maxwell’s Multipole Representation

There is a one-to-one correspondence between the *p*th-order completely symmetric tensors and the homogeneous polynomials of degree *p* in the three-dimensional [3,9]. According to Sylvester’s theorem [22], the *p*th-order deviator has the Maxwell’s multipole representation. The representation declares that the *p*th-order deviator is expressed by the tensor product of *p* unit vectors $n_{r}$ (*r* = 1, ···, *p*) multiplied by a positive scalar *A*,

(11) $$H^{\left(P\right)}=A⎣n_{1}⊗n_{2}⊗\cdots ⊗n_{p}⎦$$

where ⎣T⎦ denotes the traceless symmetric part of the tensor ***T***. The unit vectors $n_{1},n_{2},\cdots ,n_{p}$ are uniquely determined by $H^{\left(p\right)}$ within sign changes in pairs. Thus $\pm n_{1},\pm n_{2},\cdots ,\pm n_{p}$ are corresponded to 2*p* poles of a unit sphere, which are called multipole structures of deviators. This simple geometric picture was originally suggested by Maxwell [22] and further executed by Backus [23] and Baerheim [24,25]. It is worth noting that Zou and Zheng [19] provided a direct and constructive establishment of Maxwell’s multipole representation. Maxwell’s multipole representation displays a geometric image on the anisotropic structure of the deviator with its unit vector set {$n_{1},n_{2},\cdots ,n_{p}$}, so it is very useful in the tensor theory.

The symmetry groups of $H^{\left(P\right)}$ and $\epsilon H^{\left(P\right)}$ in the Equation (8) are

(12) $$\begin{array}{l} g[H^{\left(p\right)}]=\left\{Q\in O(3)|Q*H^{\left(p\right)}=H^{\left(p\right)}\right\}\Leftrightarrow \\ g[H^{\left(p\right)}]=\left\{Q\in O(3)|\left\lfloor Q*n_{1}⊗Q*n_{2}⊗\cdots ⊗Q*n_{P}\right\rfloor =\left\lfloor n_{1}⊗n_{2}⊗\cdots ⊗n_{P}\right\rfloor \right\}, \end{array}$$

(13) $$\begin{array}{l} g[\epsilon H^{\left(p\right)}]=\left\{Q\in O(3)|Q*\epsilon H^{\left(p\right)}=\epsilon H^{\left(p\right)}\right\}\Leftrightarrow \\ g[\epsilon H^{\left(p\right)}]=\left\{Q\in O(3)|\left\lfloor Q*n_{1}⊗Q*n_{2}⊗\cdots ⊗Q*n_{P}\right\rfloor =\left\lfloor n_{1}⊗n_{2}⊗\cdots ⊗n_{P}\right\rfloor ,\det(Q)=1\right\}\\ \cup \left\{Q\in O(3)|\left\lfloor Q*n_{1}⊗Q*n_{2}⊗\cdots ⊗Q*n_{P}\right\rfloor =-\left\lfloor n_{1}⊗n_{2}⊗\cdots ⊗n_{P}\right\rfloor ,\det(Q)=-1\right\} \end{array}$$

respectively. Simply put, the orthogonal transformation Q is a symmetry transformation of $H^{\left(P\right)}$ if the unit vector set {$n_{1},n_{2},\cdots ,n_{p}$} is invariant or change sign in pairs. Since the permutation tensor ϵ is hemitropic, i.e., Q*ϵ=−ϵ when det(Q)=−1, so the value of $⎣n_{1}⊗n_{2}⊗\cdots ⊗n_{p}⎦$ should change sign in Equation (13).

Based on the spatial geometric relations of the unit vectors, this paper provides an intuitive and convenient approach to reveal the symmetry axis and mirror plane of the deviator. The Cartan decomposition, by contrast, is an algebraic way used in papers [2,3,4,5,6,9,10] for the determination of symmetry types of even-type deviators. Instead of using a subgroup of SO(3) on most of the papers, the symmetry types of even-order tensor are exactly represented by a subgroup of O(3) in this paper.

## 3. Results

In this section, the possible symmetry types from formula (10) will be checked specifically through the unit vector set from Maxwell’s multipole representation of even-type deviators. Obviously, the scalar *α* only has the symmetry of $K_{h}$ and the vector ϵv only has the symmetry of $C_{\infty h}$.

### 3.1. Symmetry Types of $H^{\left(2\right)}$

**Figure 2:**
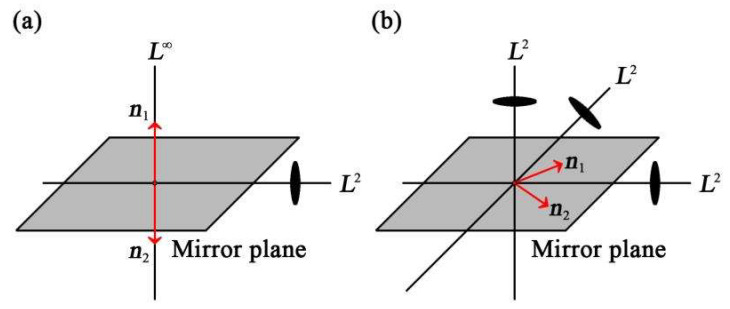
The two unit vectors and the corresponding symmetry elements: (**a**) $D_{\infty h}$ symmetry, its elements are made up of $L^{\infty }\infty L^{2}\left(\infty +1\right)PC$; (**b**) $D_{2h}$ symmetry, its elements are made up of $3L^{2}3PC$.

A second-order deviator includes a set of two unit vectors $n_{1}$ and $n_{2}$. The orthogonal transformation Q is a symmetry transformation if the two unit vectors are invariant or change sign in pairs. As shown in Figure 2, it is found that $n_{1}$ and $n_{2}$ have two distinct symmetries, namely $D_{\infty h}$ symmetry when $n_{1}//n_{2}$, and $D_{2h}$ symmetry otherwise.

### 3.2. Symmetry Types of $\epsilon H^{\left(3\right)}$

The analytical process can be summarized as Table 3: The second column indicates eight possible symmetry types of $\epsilon H^{\left(3\right)}$ from the preliminary result (10), and the third column gives the elements of the point group. By analyzing the symmetry of the three unit vectors, the possible symmetry types in the second column are checked one by one and then the accurate symmetry types of $\epsilon H^{\left(3\right)}$ is obtained. For example, the symmetry $D_{2h}$ share the same unit vector set with $T_{h}$, and $D_{2h}\subset T_{h}$, then $D_{2h}$ degenerates into $T_{h}$. There is no unit vector set with $D_{\infty h}$ symmetry, so it is inexistence. For the other six types of symmetry, the unit vector sets in the fourth column show the multipole structure of $\epsilon H^{\left(3\right)}$, the positional relation of which is also described below though the elements of point group, such as mirror plane (MP) and *n*-fold rotation axis ($L^{n}$):

(1) $C_{i}$ symmetry, three arbitrary unit vectors;
(2) $C_{3i}$ symmetry, the three unit vectors are obtained by rotating a unit vector through $\frac{2\pi }{3}$ on the $L^{3}$ axis;
(3) $D_{3d}$ symmetry, the three unit vectors are located on a plane perpendicular to the $L^{3}$ axis and share the same separation angle ($\frac{2\pi }{3}$) with each other;
(4) $C_{2h}$ symmetry, one unit vector is located on the $L^{2}$ axis, the other two unit vectors are located on the MP or take the MP as their mid-separate surface;
(5) $T_{h}$ symmetry, the three unit vectors are located on three orthogonal $L^{2}$ axes;
(6) $C_{\infty h}$ symmetry, the three unit vectors are located on the $L^{\infty }$ axis.

### 3.3. Symmetry Types of $H^{\left(4\right)}$

By a similar argument, the deviator $H^{\left(4\right)}$ was found with seven symmetry types. Because symmetries of $C_{3i},\;C_{4h},\;C_{\infty h}$ and $T_{h}$ degenerate into symmetries of $D_{3d},D_{4h},D_{\infty h}$ and $O_{h}$, respectively. The unit vector sets $\left\{n_{1},n_{2},n_{3},n_{4}\right\}$ corresponding to the seven symmetry types are listed in Table 4, specified as follows:

(1) $C_{i}$ symmetry, four arbitrary unit vectors;
(2) $D_{3d}$ symmetry, one unit vector is located on the $L^{3}$ axis, the other three unit vectors are obtained by rotating a unit vector through $\frac{2\pi }{3}$ on the $L^{3}$ axis;
(3) $C_{2h}$ symmetry, the four unit vectors are located on the MP in pair(s) or take the MP as their mid-separate surface in pair(s);
(4) $D_{2h}$ symmetry, there are two situations: (i) the four unit vectors are, respectively, located on the lateral edges of a rectangular based pyramid; (ii) the four unit vectors are located on the MP in pair(s) and take another MP as their mid-separate surface;
(5) $D_{4h}$ symmetry, there are also two situations: (i) the four unit vectors are obtained by rotating a unit vector through $\frac{\pi }{2}$ on the $L^{4}$ axis; (ii) all four unit vectors are located on the MP, which is perpendicular to the $L^{4}$ axis, and $n_{1}⊥n_{2},n_{3}⊥n_{4}$;
(6) $D_{\infty h}$ symmetry, the four unit vectors are located on the $L^{\infty }$ axis;
(7) $O_{h}$ symmetry, the four unit vectors are respectively located on the space diagonals of a cube.

### 3.4. Symmetry Types of $\epsilon H^{\left(5\right)}$

Similarly, only 10 types of symmetry exist between the preliminary 14 types of symmetry. Symmetry types of $T_{h},O_{h},I_{h}$ and $D_{\infty h}$ are unable to obtain by the set of five unit vectors. The unit vector sets $\left\{n_{1},n_{2},n_{3},n_{4},n_{5}\right\}$ corresponding to the 10 types of symmetry are listed in Table 5. The symmetry types are described as follows:

(1) $C_{i}$ symmetry, five arbitrary unit vectors;
(2) $C_{3i}$ symmetry, two unit vectors are located on the $L^{3}$ axis, three other unit vectors are obtained by rotating a unit vector through $\frac{2\pi }{3}$ on the $L^{3}$ axis;
(3) $C_{5i}$ symmetry, the five unit vectors are obtained by rotating a unit vector through $\frac{2\pi }{5}$ on the $L^{5}$ axis;
(4) $D_{3d}$ symmetry, two unit vectors are located on the $L^{3}$ axis, the other three unit vectors lie on a plane perpendicular to the $L^{3}$ axis, and they have the same separation angle ($\frac{2\pi }{3}$) to each other;
(5) $D_{5d}$ symmetry, the five unit vectors lie on a plane perpendicular to the $L^{5}$ axis, and they have the same separation angle ($\frac{2\pi }{5}$) to each other;
(6) $C_{2h}$ symmetry, one unit vector is located on the $L^{2}$ axis, the other four unit vectors lie on the MP in pair(s) or take the MP as their mid-separate surface in pair(s);
(7) $C_{4h}$ symmetry, one unit vector is located on the $L^{4}$ axis, there are two possibilities for the other four unit vectors: (i) the four unit vectors are obtained by rotating a unit vector through $\frac{\pi }{2}$ on the $L^{2}$ axis; (ii) all of the four unit vectors lie on the MP which is perpendicular to the $L^{4}$ axis, and $n_{2}⊥n_{3},n_{4}⊥n_{5}$;
(8) $D_{2h}$ symmetry, three unit vectors are located on the $L^{2}$ axis, the other two unit vectors lie on an MP and regard another MP as their mid-separate surface;
(9) $D_{4h}$ symmetry, one unit vector is located on the $L^{4}$ axis, the other four unit vectors lie on the MP which is perpendicular to the $L^{4}$ axis and the angle between the adjacent vectors is $\frac{\pi }{4}$;
(10) $C_{\infty h}$ symmetry, the five unit vectors are all located on the $L^{\infty }$ axis.

### 3.5. Symmetry Types of $H^{\left(6\right)}$

For $H^{\left(6\right)}$, only 12 types of symmetry exist between the preliminary 16 types of symmetry. Because symmetry of $C_{5i},\;C_{4h},\;C_{6h}$ and $C_{\infty h}$ degenerate into the symmetry of $D_{5d},\;D_{4h},\;D_{6h}$ and $D_{\infty h}$, respectively. The unit vector sets $\left\{n_{1},\;n_{2},\;n_{3},\;n_{4},\;n_{5},\;n_{6}\right\}$ corresponding to the 12 types of symmetry are listed in Table 6, which are described as follows:

(1) $C_{i}$ symmetry, six arbitrary unit vectors;
(2) $C_{3i}$ symmetry, the six unit vectors are obtained by rotating two different unit vectors through $\frac{2\pi }{3}$ on the $L^{3}$ axis;
(3) $D_{3d}$ symmetry, there are two situations: (i) three unit vectors are located on the $L^{3}$ axis, the other three unit vectors are obtained by rotating a unit vector through $\frac{2\pi }{3}$ on the $L^{3}$ axis; (ii) the six unit vectors are obtained by rotating two different unit vectors through $\frac{2\pi }{3}$ on the $L^{3}$ axis, and the two unit vectors are on the same MP;
(4) $D_{5d}$ symmetry, one unit vector is located on the $L^{5}$ axis, the other five unit vectors are obtained by rotating a unit vector through $\frac{2\pi }{5}$ on the $L^{5}$ axis;
(5) $C_{2h}$ symmetry, the six unit vectors lie on the MP in pair(s) or take the MP as their mid-separate surface in pair(s);
(6) $D_{2h}$ symmetry, there are two situations: (i) two unit vectors lie on an MP and take another MP as their mid-separate surface, the other four unit vectors are located on the lateral edges of a rectangular pyramid, respectively; (ii) the six unit vectors lie on an MP in pair(s) and take another MP as their mid-separate surface;
(7) $D_{4h}$ symmetry, two unit vectors are located on the $L^{4}$ axis. Two possibilities are retained in the other four unit vectors: (i) the four unit vectors are obtained by rotating a unit vector through $\frac{\pi }{2}$ on the $L^{4}$ axis; (ii) all four unit vectors lie on an MP which is perpendicular to the $L^{4}$ axis, and $n_{3}⊥n_{4},n_{5}⊥n_{6};$
(8) $D_{6h}$ symmetry, the six unit vectors are obtained by rotating a unit vector through $\frac{\pi }{3}$ on the $L^{6}$ axis;
(9) $D_{\infty h}$ symmetry, the six unit vectors are located on the $L^{\infty }$ axis;
(10) $T_{h}$ symmetry, the six unit vectors are located on the face diagonals of a cube, respectively;
(11) $O_{h}$ symmetry, the six unit vectors are located on three orthogonal $L^{4}$ axes in pair, respectively;
(12) $I_{h}$ symmetry, the six unit vectors are located on six $L^{5}$ axes, respectively.

### 3.6. Characteristic Web Trees

**Figure 3:**
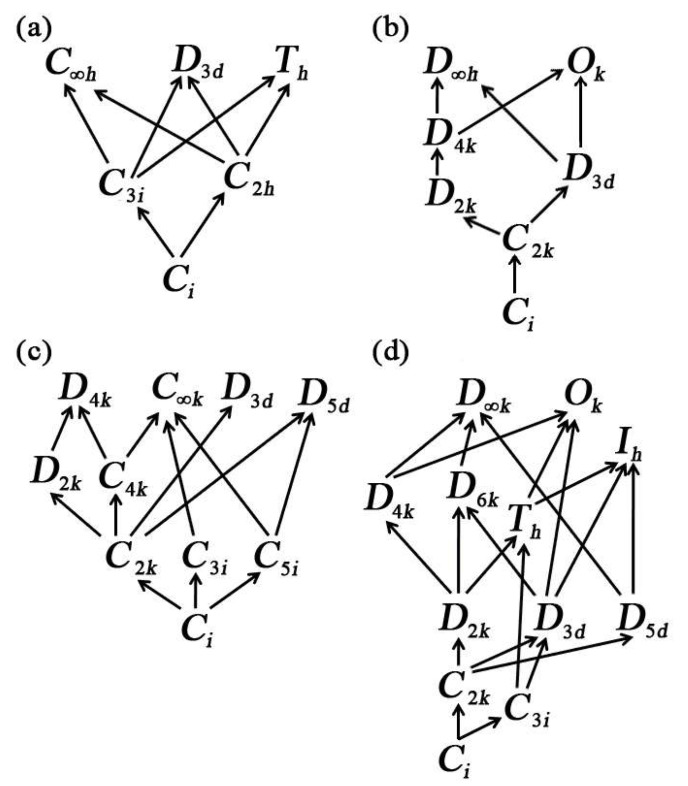
Characteristic web trees: (**a**)$\;\epsilon H^{\left(3\right)}$; (**b**) $H^{\left(4\right)}$^)^; (**c**) $\epsilon H^{\left(5\right)}$; (**d**) $H^{\left(6\right)}$.

The examinations of the integrity of the unit vector sets in the Table 3, Table 4, Table 5 and Table 6 presented above are necessary. This is accomplished by introducing the characteristic web tree of tensors [12]. It is known that the symmetry group may contain some other symmetry groups, so it has a relatively higher symmetry. For two symmetry groups *A* and *B*, if *A* ⊂ *B*, then *A* is called the subgroup of *B*, while *B* is the mother group of *A*. If the other mother groups contained by *B* are not obtained by *A*, then *A→B* is defined. In so doing, all possible consequences finally generate a characteristic web tree of deviators shown in Figure 3. With the insertion of an additional orthogonal transformation, the number of independent variables in the deviator may be remarkably reduced. The corresponding unit vector sets are also specialized. Take the $\epsilon H^{\left(3\right)}$ as an example, when it contains $C_{3i}$ symmetry, the unit vector set is $\left\{n\left(\theta ,\varphi +\frac{2k\pi }{3}\right),k=0,1,2\right\}$. From its characteristic web tree in Figure 3a, the following conclusions are given.

(1) $C_{3i}\;$⊂$\;T_{h}$. For $\sin\left(\theta \right)=\frac{\sqrt{6}}{3}$ and through a proper rotation, the set $\left\{n\left(\theta ,\varphi +\frac{2k\pi }{3}\right),k=0,1,2\right\}$ is the same with the set $\left\{e,n\left(\frac{\pi }{2},\varphi +\frac{k\pi }{2}\right),k=0,1\right\}$ corresponding to $T_{h}$;
(2) $C_{3i}$ ⊂ $D_{3d}$. For $\theta =\frac{\pi }{2}$, the set $\left\{n\left(\theta ,\varphi +\frac{2k\pi }{3}\right),k=0,1,2\right\}$ becomes $\left\{n\left(\frac{\pi }{2},\varphi +\frac{2k\pi }{3}\right),k=0,1,2\right\}$ corresponding to $D_{3d}$;
(3) $C_{3i}$ ⊂ $C_{\infty h}$. For θ=0, the set $\left\{n\left(\theta ,\varphi +\frac{2k\pi }{3}\right),k=0,1,2\right\}$ becomes {e,e,e}, which is corresponding to $C_{\infty h}$.

Additionally, this relation could be checked for each pair of symmetry groups in Figure 3. Namely, if *A* ⊂ *B*, by introducing some constraint conditions and commencing a proper rotation, the unit vector set corresponding to *A* will become the unit vector set corresponding to *B*. According to such an examination, the correctness and integrity of the unit vector sets in Table 3, Table 4, Table 5 and Table 6 are verified.

### 3.7. The General Results

For higher order deviator $H_{e}^{\left(n\right)}\;\left(n\geq 7\right)$, its symmetry types are determined by the unit vector set of similar methods. The preliminary symmetry types of (10) will be checked one by one through the set unit vectors. For symmetry type A, there are three possible situations: (1) the unit vector sets with symmetry A are found out and the other relatively higher symmetries are not possessed by them, then the symmetry A is proved to exist; (2) these unit vector sets possess a relatively higher symmetry B, namely A ⊂ B, which means that symmetry A degenerates into symmetry B and symmetry A is nonexistent; (3) the unit vector set cannot be found out, then symmetry A is nonexistent. The preliminary symmetry types of $H_{e}^{\left(n\right)}\;\left(n\geq 7\right)$ in (10) are analyzed as below:

(1) It is obvious that $C_{i},\;C_{2h}$ and $D_{2h}$ symmetries exist.
(2) For $C_{3i},\;C_{5i},\;\cdots ,\;C_{ki}$, there are three situations when *k* is odd and k≤n: (i) The symmetry $C_{ki}$ exists when n is odd (namely $\epsilon H^{\left(n\right)}$). One of the unit vector sets is: the (*n* − *k*) unit vectors are located on the $L^{k}$ axis and the other *k* unit vectors are obtained by rotating a unit vector through $\frac{2\pi }{k}$ on the $L^{k}$ axis; (ii) the symmetry $C_{ki}$ exists when n is even (namely $H^{\left(n\right)}$) and $k\leq \frac{n}{2}$. One of the unit vector sets is: the (*n* − 2*k*) unit vectors are located on the $L^{k}$ axis, the other 2*k* unit vectors are obtained by rotating two different unit vectors through $\frac{2\pi }{k}$ on the $L^{k}$ axis; (iii) the symmetry $C_{ki}$ is nonexistent when *n* is even and $k>\frac{n}{2}$. The unit vector set with $C_{ki}$ symmetry is: the (*n* − *k*) unit vectors are located on the $L^{k}$ axis, the other *k* unit vectors are obtained by rotating a unit vector through $\frac{2\pi }{k}$ on the $L^{k}$ axis. Meanwhile, this unit vector set owns the $L^{2}$ symmetric axis, which is perpendicular to the $L^{k}$ axis. So, symmetry $C_{ki}$ degenerates into symmetry $D_{kd}$.
(3) For $D_{3d},\;D_{5d},\;D_{7d},\;\cdots ,\;D_{kd}$, there are two situations when *k* is odd and k≤n: (i) the symmetry $D_{kd}$ exists when *n* is odd. One of the unit vector sets is: the (*n* − *k*) unit vectors are located on the $L^{k}$ axis, the other *k* unit vectors are located on a plane perpendicular to the $L^{k}$ axis and share the same separation angle $\frac{2\pi }{k}$ with each other; (ii) the symmetry $D_{kd}$ exists when *n* is even. One of the unit vector sets is: the (*n* − *k*) unit vectors are located on the $L^{k}$ axis, the other *k* unit vectors are obtained by rotating a unit vector through $\frac{2\pi }{k}$ on the $L^{k}$ axis.
(4) For $C_{4h},\;C_{6h},\;\cdots ,\;C_{kh}$, there are three situations when *k* is even and k≤n: (i) the symmetry $C_{kh}$ exists when *n* is odd. One of the unit vector sets is: the (*n* − *k*) unit vectors are located on the $L^{k}$ axis, the other *k* unit vectors are obtained by rotating a unit vector through $\frac{2\pi }{k}$ on the $L^{k}$ axis; (ii) the symmetry $C_{kh}$ exists when *n* is even and $k\leq \frac{n}{2}$. One of the unit vector sets is: the (*n* − 2*k*) unit vectors are located on the $L^{k}$ axis, the other 2*k* unit vectors are obtained by rotating two different unit vectors through $\frac{2\pi }{k}$ on the $L^{k}$ axis; (iii) the symmetry $C_{kh}$ is inexistence when *n* is even and $k>\frac{n}{2}$. The unit vector set with $C_{kh}$ symmetry is: the (*n* − *k*) unit vectors are located on the $L^{k}$ axis, the other *k* unit vectors are obtained by rotating a unit vector through $\frac{2\pi }{k}$ on the $L^{k}$ axis. Meanwhile, this unit vector set owns the $L^{2}$ symmetric axis, which is perpendicular to the $L^{k}$ axis, so symmetry $C_{kh}$ degenerates into symmetry $D_{kh}$.
(5) For $D_{4h},\;D_{6h},\;\cdots ,\;D_{kh}$, there are two situations when *k* is even and k≤n: (i) the symmetry $D_{kh}$ exists when *n* is odd. One of the unit vector sets is: the (*n* − *k*) unit vectors are located on the $L^{k}$ axis, the other *k* unit vectors are located on the MP, which is perpendicular to the $L^{k}$ axis and the angle between adjacent vectors is $\frac{\pi }{k}$; (ii) symmetry $D_{kh}$ exists when *n* is even. One of the unit vector sets is: the (*n* − *k*) unit vectors are located on the $L^{k}$ axis, the other *k* unit vectors are obtained by rotating a unit vector through $\frac{2\pi }{k}$ on the $L^{k}$ axis.
(6) Symmetry $C_{\infty h}$ exists and $D_{\infty h}$ is nonexistent when *n* is odd; instead, the symmetry $D_{\infty h}$ exists and $C_{\infty h}$ is nonexistent when *n* is even. The *n* unit vectors are all located on the $L^{\infty }$ axis in both cases.
(7) For $T_{h}$ and $O_{h}$, clearly that the two point groups, respectively, described the geometric symmetry of a regular tetrahedron and a cube, and $T_{h}\subset O_{h}$ is just like the regular tetrahedron embedded inside the cube. According to the previous results, the $\epsilon H^{\left(3\right)}$ has symmetry $T_{h}$ when its three unit vectors are on three concurrent edges of a cube. Additionally, the $H^{\left(6\right)}$ also has symmetry $T_{h}$ when the six unit vectors are on face diagonals (in adjacent three faces) of a cube. Notice that $\epsilon H^{\left(3\right)}$ and $H^{\left(6\right)}$ are unable to obtain symmetry $O_{h}$ because their value will change sign for these two corresponding unit vector sets. The symmetry $O_{h}$ is obtained by $H^{\left(4\right)}$ when the four unit vectors are on space diagonals of a cube. Based on the similar principle of doing intersection of point groups and two negatives make an affirmative, the situations of symmetry $T_{h}$ and $O_{h}$ are as below:
  (i) When $n=3m_{1}+6m_{2}+4m_{3}$ ($m_{1}$, $m_{2}$ and $m_{3}$ are non-negative integers) and $m_{1}+6m_{2}=\mathrm{odd}$, namely *n* = 7 or n≥9, the symmetry $T_{h}$ exists for $H_{e}^{\left(n\right)}$. The *n* unit vectors are all located in a cube: $3m_{1}$ unit vectors are evenly located on three concurrent edges, $6m_{2}$ unit vectors are evenly located on six face diagonals and $4m_{3}$ unit vectors are evenly located on four space diagonals;
  (ii) When $n=3m_{1}+6m_{2}+4m_{3}$ and $m_{1}+6m_{2}=\mathrm{even}$, namely *n* = 8, 9, 10 or n≥12, the symmetry $O_{h}$ exists for $H_{e}^{\left(n\right)}$ and all of the *n* unit vectors are also located in a cube in the situation mentioned above. The reason of $m_{1}+6m_{2}=\mathrm{even}$ is that the value of $H_{e}^{\left(n\right)}$ will be invariant under even times of change in the sign.
(8) For symmetry $I_{h}$, this point group describes the geometric symmetry of a regular dodecahedron. The six $L^{5}$ axes are the lines that come through the body-centered point and two face-centered points (12 regular pentagonal faces). The ten $L^{3}$ axes are the lines that come through the body-centered point and two vertices (20 vertices). The fifteen $L^{2}$ axes are all parallel to the edges (30 edges). The deviators of $H^{\left(6\right)},\;H^{\left(10\right)}$ and $\epsilon H^{\left(15\right)}$ all contain symmetry $I_{h}$, and their sets of unit vectors are on the rotation-axes of a regular dodecahedron: the six unit vectors of $H^{\left(6\right)}$ are on six $L^{5}$ axes; the ten unit vectors of $H^{\left(10\right)}$ are on ten $L^{3}$ axes; the fifteen unit vectors of $\epsilon H^{\left(15\right)}$ are on fifteen $L^{2}$ axes. So, $H_{e}^{\left(n\right)}$ has symmetry $I_{h}$ when $n=6m_{1}+10m_{2}+15m_{3}$, namely n=6,10,12,15,16,18 or n≥20, and n≠23,29.

Above all, the general results about symmetry types of all order even-type deviators are provided and they can be stated as below:

**Theorem** **1.** *For an even-type deviator $H_{e}^{\left(n\right)}$, the symmetry types are given as follow:*

1. *$\;C_{i}\;\mathrm{for}\;n\geq 3;$*
2. *$\;C_{3i},\;C_{5i},\;\cdots ,\;C_{\left(2k-1\right)i}\;\mathrm{for}\;\{\begin{array}{c} 2k-1\leq n,\;\mathrm{when}\;n\;\mathrm{is}\;\mathrm{odd},\\ 2k-1\leq \frac{n}{2},\;\mathrm{when}\;n\;\mathrm{is}\;\mathrm{even}; \end{array}$*
3. *$\;D_{3d},\;D_{5d},\;D_{7d},\;\cdots ,\;D_{\left(2k-1\right)d}\;\mathrm{for}\;3\leq 2k-1\leq n$*
4. *$\;C_{2h},\;C_{4h},\;\cdots ,\;C_{\left(2k\right)h}\;\mathrm{for}\;\{\begin{array}{c} 2k\leq n,\;\mathrm{when}\;n\;\mathrm{is}\;\mathrm{odd},\\ 2k\leq \frac{n}{2},\;\mathrm{when}\;n\;\mathrm{is}\;\mathrm{even}; \end{array}$*
5. *$\;D_{2h}\;\mathrm{for}\;n\geq 2\;\mathrm{and}\;n\neq 3$;*
6. *$\;D_{4h},\;D_{6h},\;\cdots ,\;D_{\left(2k\right)h}\;\mathrm{for}\;4\leq 2k\leq n$;*
7. *$\;C_{\infty h}\;\mathrm{for}\;n\;\mathrm{is}\;\mathrm{odd};\;$*
8. *$\;D_{\infty h}\;\mathrm{for}\;n\;\mathrm{is}\;\mathrm{even},\;\mathrm{and}\;n\neq 0;\;$*
9. *$\;T_{h}\;\mathrm{for}\;n=3,6,7,\;\mathrm{or}\;n\geq 9;\;$*
10. *$\;O_{h}\;\mathrm{for}\;n=4,6,8,9,10,\;\mathrm{or}\;n\geq 12;\;$*
11. *$\;I_{h}\;\mathrm{for}\;n=6,10,12,15,16,18,\;\mathrm{or}\;n\geq 20,\;\mathrm{and}\;n\neq 23,29;$*
12. *$\;K_{h}\;\mathrm{for}\;n=0,\;\mathrm{namely}\;\mathrm{scalar}.\;$*

## 4. Conclusions

In this paper, the symmetry types of all even-type deviators have been derived by the idea of exclusion of two steps: Firstly, the preliminary symmetry types are obtained by doing an exclusion towards all possible symmetry types, which is according to the order of tensor and the existing results of the literature review. Secondly, the symmetry types of *p*-order deviator $H_{e}^{\left(p\right)}$ are determined by analyzing its unit vector set $\left\{n_{1},n_{2},\cdots ,n_{p}\right\}$ under the orthogonal transformation, which is Maxwell’s multipole representation of deviator. Based on the spatial geometric relations of the unit vectors, an intuitive and convenient approach is provided to reveal the potential symmetric axes and the mirror planes of the deviator. By comparing the results of Ihrig and Golubitsky [16] (Theorem 6.6), Olive and Auffray [15] (Theorem 5.1), some modifications are made: (1) $D_{2h}$ is not a symmetry type for $\varepsilon H^{\left(3\right)}$; (2) $T_{h}$ is a symmetry type for $\varepsilon H^{\left(3\right)}$; (3) $O_{h}$ is not a symmetry type for $\varepsilon H^{\left(3\right)}$; (4) $I_{h}$ is not a symmetry type for ***H***^(16)^.

Maxwell’s multipole representation displays a geometric image on the anisotropic structure of the deviator with its unit vector set, so it is very useful in the tensor theory. For each symmetry type of the even-type deviator up to sixth-order, this paper gives the corresponding unit vector set with the representation of a specific multipole structure. Another important application on symmetry problem is that the multipole structure can be used in symmetry identification of an unknown physical tensor and for necessary back-calculation of the involved physical coefficients. The integrity of all involved unit vector sets has been checked through the characteristic web tree.

The symmetry classification of even-type deviators is the basis for the symmetry problems of an arbitrary even-order physical tensor. Application examples are given in the Appendix A according to the method and results of this paper for all kinds of fourth-order tensors, such as elasticity tensor, flexoelectric tensor and photo-elastic tensor. The symmetry classification of these tensors has already been studied in different literatures, and all these related literatures were discussed through a similar computational method, which has the drawback of not providing general results. Furthermore, the complexity of the symmetry problem increases as the order of the tensor. This paper provides the general results about symmetry types of all order even-type deviators. As the follow-up studies, a complete answer to the symmetry types of even-order tensors also can be realized with the similar exclusion of two steps: Firstly, the preliminary symmetry types could be applied to all general tensors; secondly, the accurate exclusion will be achieved by doing the intersection of point group instead. Details of this process are exhibited by giving examples in Appendix A. The method and investigation of this paper can also be extended to the situation of odd-order tensor without any increase of complexity.

## Figures and Tables

**Table 1:** The meaning of symbols in this paper.

Symbol	The Meaning
***C***	The elasticity tensor
O(3)	The max orthogonal group
***T*** ^(*n*)^	*n*th-order general tensor
*δ*	The Kronecker symbol
*g*	The symmetry group
***Q***	The orthogonal tensor
***H*** ^(*n*)^	*n*th-order deviator
**ϵ**	The permutation tensor
$H_{e}^{\left(n\right)}$	*n*th-order even-type deviator
$H_{o}^{\left(n\right)}$	*n*th-order odd-type deviator

**Table 2:** The total collection of O(3)-closed subgroups [18].

Physical Classes (*L* = Integer ≥ 1)	No.	International Symbol	Schoenflies Symbol	Order of Group
Triclinic	1	I	$C_{1}$	1
	2	$\bar{I}$	$C_{i}$	2
Monoclnic	3	2	$C_{2}$	2
	4	m	$C_{1h}$	2
	5	2/m	$C_{2h}$	4
Orthorhombic	6	222	$D_{2}$	4
	7	mm2	$C_{2v}$	4
	8	mmm	$D_{2h}$	8
4*L*-gonal (*n* = 4*L*)	9	n	$C_{n}$	*n*
	10	$\bar{n}$	$C_{\left(n/2\right)i}$	*n*
	11	n/m	$C_{nh}$	2*n*
	12	n22	$D_{n}$	2*n*
	13	nmm	$C_{nv}$	2*n*
	14	$\bar{n}m$	$D_{\left(n/2\right)d}$	2*n*
	15	n/mmm	$D_{nh}$	4*n*
(2*L* + 1)-gonal (*n* = 2*L +* 1)	16	n	$C_{n}$	*n*
	17	$\bar{n}$	$C_{ni}$	2*n*
	18	n2	$D_{n}$	2*n*
	19	nm	$C_{nv}$	2*n*
	20	$\bar{n}\;m$	$D_{nd}$	4*n*
(4*L* + 2)-gonal (*n* = 4*L +* 2)	21	n	$C_{n}$	*n*
	22	$\bar{n}$	$C_{\left(n/2\right)h}$	*n*
	23	n/m	$C_{nh}$	2*n*
	24	n22	$D_{n}$	2*n*
	25	nmm	$C_{nv}$	2*n*
	26	$\bar{n}2m$	$D_{\left(n/2\right)h}$	2*n*
	27	n/mmm	$D_{nh}$	4*n*
Cubic	28	23	*T*	12
	29	$m\bar{3}$	$T_{h}$	24
	30	432	*O*	24
	31	$\bar{4}32$	$T_{d}$	24
	32	$m\bar{3}m$	$O_{h}$	48
Icosahedral	33	235	*I*	60
	34	$m\bar{35}$	$I_{h}$	120
Cylindrical	35	∞	$C_{\infty }$, or SO(2)	∞
	36	$\bar{\infty }$	$C_{\infty h}$	∞
	37	∞2	$D_{\infty }$, or O(2)	∞
	38	∞2	$C_{\infty v}$	∞
	39	∞m	$D_{\infty h}$	∞
Spherical	40	2∞	*K*, or SO(3)	∞
	41	$2m\bar{\infty }$	$K_{h}$, or O(3)	∞

**Table 3:** Symmetry types and the unit vector sets of $\epsilon H^{\left(3\right)}$. Note (similarly hereinafter): the symmetry groups marked in red and inside parentheses degenerate or simply do not exist, Null means that there are no invariant unit vector sets under this point group. For simplicity, the principal axis of the point group is set to the ***e***-axis).

No.	Symmetry Type	Elements	Set of Unit Vector
1	$C_{i}$	C	$\left\{n_{1},n_{2},n_{3}\right\}$
2	$C_{3i}$	$L^{3}C$	$\left\{n\left(\theta ,\varphi +\frac{2k\pi }{3}\right),k=0,1,2\right\}$
3	$D_{3d}$	$L^{3}3L^{2}3PC$	$\left\{n\left(\frac{\pi }{2},\varphi +\frac{2k\pi }{3}\right),k=0,1,2\right\}$
4	$C_{2h}$	$L^{2}PC$	$\left\{e,n\left(\frac{\pi }{2},\varphi_{1}\right),n\left(\frac{\pi }{2},\varphi_{2}\right)\right\}$ or {e,n(θ,φ),n(θ,φ+π)}
5	$\left(D_{2h}\right)$, $T_{h}$	$3L^{2}4L^{3}3PC$	$\left\{e,n\left(\frac{\pi }{2},\varphi +\frac{k\pi }{2}\right),k=0,1\right\}$
6	$C_{\infty h}$	$L^{\infty }PC$	{e,e,e}
	$\left(D_{\infty h}\right)$		Null

**Table 4:** Symmetry types and the unit vector sets of $H^{\left(4\right)}$.

No.	Symmetry Type	Elements	Set of Unit Vector
1	$C_{i}$	C	$\left\{n_{1},n_{2},n_{3},n_{4}\right\}$
2	$(C_{3i}),D_{3d}$	$L^{3}3L^{2}3PC$	$\left\{e,n\left(\theta ,\varphi +\frac{2k\pi }{3}\right),k=0,1,2\right\}$
3	$C_{2h}$	$L^{2}PC$	$\left\{S_{1},S_{2}\right\}$ $S_{i}=\left\{n\left(\frac{\pi }{2},\varphi_{i1}\right),n\left(\frac{\pi }{2},\varphi_{i1}\right)\right\}$ $\;\mathrm{or}\;\left\{n\left(\theta_{i},\varphi_{i}\right),n\left(\theta_{i},\varphi_{i}+\pi \right)\right\}$
4	$D_{2h}$	$3L^{2}3PC$	{n(θ,φ),n(θ,π−φ),n(θ,π+φ),n(θ,2π−φ)} **or** $\left\{S_{1},S_{2}\right\}$ $S_{i}=\left\{n\left(\frac{\pi }{2},\varphi_{i}\right),n\left(\frac{\pi }{2},\pi -\varphi_{i}\right)\right\}$ $\mathrm{or}\left\{n\left(\theta_{i},0\right),n\left(\theta_{i},\pi \right)\right\}\;\mathrm{or}\left\{n\left(\theta_{i},\frac{\pi }{2}\right),n\left(\theta_{i},\frac{3\pi }{2}\right)\right\}$
5	$(C_{4h}),D_{4h}$	$L^{4}4L^{2}5PC$	$\left\{n\left(\theta ,\varphi +\frac{k\pi }{2}\right),k=0,1,2,3\right\}$ $\mathrm{or}\;\left\{n\left(\frac{\pi }{2},\varphi_{1}\right),n\left(\frac{\pi }{2},\varphi_{1}+\frac{\pi }{2}\right),n\left(\frac{\pi }{2},\varphi_{2}\right),n\left(\frac{\pi }{2},\varphi_{2}+\frac{\pi }{2}\right)\right\}$
6	$(C_{\infty h}),D_{\infty h}$	$L^{\infty }\infty L^{2}\left(\infty +1\right)PC$	{e,e,e,e}
7	$(T_{h}),O_{h}$	$3L^{4}4L^{3}6L^{2}9PC$	$\left\{\frac{e+m_{1}+m_{2}}{\sqrt{3}},\frac{e-m_{1}+m_{2}}{\sqrt{3}},\frac{e+m_{1}-m_{2}}{\sqrt{3}},\frac{e-m_{1}-m_{2}}{\sqrt{3}}\right\}$ $m_{1}=n\left(\frac{\pi }{2},0\right)$, $\;m_{2}=n\left(\frac{\pi }{2},\frac{\pi }{2}\right)$

**Table 5:** Symmetry types and the unit vector sets of $\epsilon H^{\left(5\right)}$.

No.	Symmetry Type	Elements	Set of Unit Vector
1	$C_{i}$	C	$\left\{n_{1},n_{2},n_{3},n_{4},n_{5}\right\}$
2	$C_{3i}$	$L^{3}C$	$\left\{e,e,n\left(\theta ,\varphi +\frac{2k\pi }{3}\right),k=0,1,2\right\}$
3	$C_{5i}$	$L^{5}C$	$\left\{n\left(\theta ,\varphi +\frac{2k\pi }{5}\right),k=0,1,2,3,4\right\}$
4	$D_{3d}$	$L^{3}3L^{2}3PC$	$\left\{e,e,n\left(\frac{\pi }{2},\varphi +\frac{2k\pi }{3}\right),k=0,1,2\right\}$
5	$D_{5d}$	$L^{5}5L^{2}5PC$	$\left\{n\left(\frac{\pi }{2},\varphi +\frac{2k\pi }{5}\right),k=0,1,2,3,4\right\}$
6	$C_{2h}$	$L^{2}PC$	$\left\{e,S_{1},S_{2}\right\}$ $S_{i}=\left\{n\left(\frac{\pi }{2},\varphi_{i1}\right),n\left(\frac{\pi }{2},\varphi_{i2}\right)\right\}\;$ $\mathrm{or}\;\left\{n\left(\theta_{i},\varphi_{i}\right),n\left(\theta_{i},\varphi_{i}+\pi \right)\right\}$
7	$C_{4h}$	$L^{4}PC$	$\left\{e,n\left(\theta ,\varphi +\frac{k\pi }{2}\right),k=0,1,2,3\right\}$ $\mathrm{or}\;\left\{e,n\left(\frac{\pi }{2},\varphi_{1}\right),n\left(\frac{\pi }{2},\varphi_{1}+\frac{\pi }{2}\right),n\left(\frac{\pi }{2},\varphi_{2}\right),n\left(\frac{\pi }{2},\varphi_{2}+\frac{\pi }{2}\right)\right\}$
8	$D_{2h}$	$3L^{2}3PC$	$\left\{S,e,n\left(\theta ,\varphi +\frac{k\pi }{2}\right),k=0,1\right\}$ $S=\left\{n\left(\frac{\pi }{2},\varphi_{1}\right),n\left(\frac{\pi }{2},\pi -\varphi_{1}\right)\right\}$ or $\left\{n\left(\theta_{1},0\right),n\left(\theta_{1},\pi \right)\right\}$ or $\left\{n\left(\theta_{1},\frac{\pi }{2}\right),n\left(\theta_{1},\frac{3\pi }{2}\right)\right\}$
9	$D_{4h}$	$L^{4}4L^{2}5PC$	$\left\{e,n\left(\frac{\pi }{2},\varphi +\frac{k\pi }{4}\right),k=0,1,2,3\right\}$
10	$C_{\infty h}$	$L^{\infty }PC$	{e,e,e,e,e}
	$\left(T_{h},O_{h},I_{h},D_{\infty h}\right)$		Null

**Table 6:** Symmetry types and the unit vector sets of **H**^(6)^.

No.	Symmetry Type	Elements	Set of Unit Vector
1	$C_{i}$	C	$\left\{n_{1},n_{2},n_{3},n_{4},n_{5},n_{6}\right\}$
2	$C_{3i}$	$L^{3}C$	$\left\{n\left(\theta_{1},\varphi_{1}+\frac{2k\pi }{3}\right),n\left(\theta_{2},\varphi_{2}+\frac{2k\pi }{3}\right),k=0,1,2\right\}$
3	$D_{3d}$	$L^{3}3L^{2}3PC$	$\left\{e,e,e,n\left(\theta ,\varphi +\frac{2k\pi }{3}\right),k=0,1,2\right\}$ or $\left\{n\left(\theta_{1},\varphi +\frac{2k\pi }{3}\right),n\left(\theta_{2},\varphi +\frac{2k\pi }{3}\right),k=0,1,2\right\}$
4	$(C_{5i}),D_{5d}$	$L^{5}5L^{2}5PC$	$\left\{e,n\left(\theta ,\varphi +\frac{2k\pi }{5}\right),k=0,1,2,3,4\right\}$
5	$C_{2h}$	$L^{2}PC$	$\left\{S_{1},S_{2},S_{3}\right\}$ $S_{i}=\left\{n\left(\frac{\pi }{2},\varphi_{i1}\right),n\left(\frac{\pi }{2},\varphi_{i2}\right)\right\}\;$ $\mathrm{or}\;\left\{n\left(\theta_{i},\varphi_{i}\right),n\left(\theta_{i},\varphi_{i}+\pi \right)\right\}$
6	$D_{2h}$	$3L^{2}3PC$	$\left\{S_{1},n\left(\theta ,\varphi \right),n\left(\theta ,\pi -\varphi \right),n\left(\theta ,\pi +\varphi \right),n\left(\theta ,2\pi -\varphi \right)\right\}or\left\{S_{1},S_{2},S_{3}\right\}$ $S_{i}=\;\left\{n\left(\frac{\pi }{2},\varphi_{i}\right),n\left(\frac{\pi }{2},\pi -\varphi_{i}\right)\right\}$ or $\left\{n\left(\theta_{i},0\right),n\left(i,\pi \right)\right\}$ or $\left\{n\left(\theta_{i},\frac{\pi }{2}\right),n\left(\theta_{i},\frac{3\pi }{2}\right)\right\}$
7	$(C_{4h}),D_{4h}$	$L^{4}4L^{2}5PC$	$\left\{e,e,n\left(\theta ,\varphi +\frac{k\pi }{2}\right),k=0,1,2,3\right\}$ or $\left\{e,e,n\left(\frac{\pi }{2},\varphi_{1}\right),n\left(\frac{\pi }{2},\varphi_{1}+\frac{\pi }{2}\right),n\left(\frac{\pi }{2},\varphi_{2}\right),n\left(\frac{\pi }{2},\varphi_{2}+\frac{\pi }{2}\right)\right\}$
8	$(C_{6h}),D_{6h}$	$L^{6}6L^{2}7PC$	$\left\{n\left(\theta ,\varphi +\frac{k\pi }{2}\right),k=0,1,2,3,4,5\right\}$
9	$(C_{\infty h}),D_{\infty h}$	$L^{\infty }\infty L^{2}\left(\infty +1\right)PC$	{e,e,e,e,e,e}
10	$T_{h}$	$3L^{2}4L^{3}3PC$	$\left\{\begin{array}{c} \frac{e+m_{1}}{\sqrt{2}},\frac{e-m_{1}}{\sqrt{2}},\frac{e+m_{2}}{\sqrt{2}},\frac{e-m_{2}}{\sqrt{2}}\\ ,\frac{m_{1}+m_{2}}{\sqrt{2}},\frac{m_{1}-m_{2}}{\sqrt{2}} \end{array}\right\}$ $m_{1}=n\left(\frac{\pi }{2},0\right)$, $\;m_{2}=n\left(\frac{\pi }{2},\frac{\pi }{2}\right)$
11	$O_{h}$	$3L^{4}4L^{3}6L^{2}9PC$	$\left\{e,e,n\left(\frac{\pi }{2}+0\right),n\left(\frac{\pi }{2}+0\right),n\left(\frac{\pi }{2}+\frac{\pi }{2}\right),n\left(\frac{\pi }{2}+\frac{\pi }{2}\right)\right\}$
12	$I_{h}$	$15L^{2}10L^{3}6L^{5}15PC$	$\left\{e,n\left(\theta ,\varphi +\frac{2k\pi }{5}\right),k=0,1,2,3,4,\theta =\arctan\left(2\right)\right\}$

## Data Availability

Data available on request due to restrictions like data capacity. The data presented in this study are available on request from the corresponding author.

## Appendix A

By using the analytical methods and results of this paper, the number and types of symmetry for all kinds of fourth-order tensors is restudied, namely the physical tensors of elasticity tensor ***C***, flexoelectric tensor ***F*** and photoelastic tensor ***M***. For the general tensor $T^{\left(4\right)}$, the possible symmetry types given by (10) are

(A1) $$C_{i},\;C_{3i},\;D_{3d},\;C_{2h},\;C_{4h},\;C_{\infty h},D_{2h},\;D_{4h},\;D_{\infty h},\;T_{h},\;O_{h},\;K_{h}$$

Similarly, the 12 symmetry types could be further refined. Considering index symmetry of the three different fourth-order physical tensors, their irreducible decomposition is

(A2) $$C=\sum_{j=1}^{2}\alpha_{j}⊕\sum_{j=1}^{2}H_{j}^{\left(2\right)}⊕H^{\left(4\right)},$$

(A3) $$F=\sum_{j=1}^{2}\alpha_{j}⊕\sum_{j=1}^{3}v_{j}⊕\sum_{j=1}^{4}H_{j}^{\left(2\right)}⊕\sum_{j=1}^{2}H_{j}^{\left(3\right)}⊕H^{\left(4\right)},$$

(A4) $$F=\sum_{j=1}^{2}\alpha_{j}⊕v⊕\sum_{j=1}^{4}H_{j}^{\left(2\right)}⊕H^{\left(3\right)}⊕H^{\left(4\right)}.$$

Then, their symmetry types are obtained through the intersection of the symmetry of the relevant deviators. From the results of this paper, the symmetry types of the relevant even-type deviators are

(A5) $$\alpha :\;K_{h};\;\epsilon v:\;C_{\infty h};\;H^{\left(2\right)}:\;D_{2h},D_{\infty H};\;\epsilon H^{\left(3\right)}:\;C_{i},\;C_{3i},\;D_{3d},\;C_{2h},\;C_{\infty h},\;T_{h};\;H^{\left(4\right)}:\;C_{i},\;D_{3d},\;C_{2h},D_{2h},\;D_{4h},\;D_{\infty h},O_{h}.$$

Thus, the results in Table A1 are obtained quickly with the complete analysis process stated as follows:

(1) The 12 possible symmetry types are listed in the second column. Corresponding to each symmetry type, the symmetry types of deviators are discovered and listed in the third to the sixth columns. Null means it is nonexistent.
(2) Considering the irreducible decomposition (A2)–(A4), this step is to check whether each symmetry type in the second column can be determined through the intersection of the symmetry of the relevant deviators. If the symmetry types exist, the number of distinct components will be given in the next step. Null is given for the other situations.
(3) Corresponding to each symmetry type of deviators, the number of distinct components is calculated due to its multipole structure (namely the unit vector sets). Thus, the number of distinct components *φ* for the three different physical tensors is calculated as:
  (A6) $$\varphi =j_{0}+j_{1}\times \varphi \left(\epsilon v\right)+j_{2}\times \varphi \left(H^{\left(2\right)}\right)+j_{3}\times \varphi \left(\epsilon H^{\left(3\right)}\right)+\varphi \left(H^{\left(4\right)}\right).$$
  where $j_{s}$ is the number of *s*th-order deviators in the irreducible decomposition. The correctness of these results in Table A1 is verified, the elasticity tensor ***C*** is referred to the article [2] and the flexoelectric tensor ***F*** is referred to the article [10]. It’s worth noting that this method could also be applied for higher even-order tensors.

**Table A1:** Symmetry types and distinct components of fourth-order tensors.

No.	Symmetry Types	g[α]	g[ϵv]	$g\left[H^{\left(2\right)}\right]$	$g\left[\epsilon H^{\left(3\right)}\right]$	$g\left[H^{\left(4\right)}\right]$	*C*	*F*	*M*
1	$C_{i}$	$K_{h}$	$C_{\infty h}$	$D_{2h}$	$C_{i}$	$C_{i}$	21	54	36
2	$C_{3i}$	$K_{h}$	$C_{\infty h}$	$D_{\infty h}$	$C_{3i}$	$D_{3d}$	Null	18	12
3	$D_{3d}$	$K_{h}$	Null	$D_{\infty h}$	$D_{3d}$	$D_{3d}$	6	10	8
4	$C_{2h}$	$K_{h}$	$C_{\infty h}$	$D_{2h}$	$C_{2h}$	$C_{2h}$	13	28	20
5	$C_{4h}$	$K_{h}$	$C_{\infty h}$	$D_{\infty h}$	$C_{\infty h}$	$D_{4h}$	Null	14	10
6	$C_{\infty h}$	$K_{h}$	$C_{\infty h}$	$D_{\infty h}$	$C_{\infty h}$	$D_{\infty h}$	Null	12	8
7	$D_{2h}$	$K_{h}$	Null	$D_{2h}$	$T_{h}$	$D_{2h}$	9	15	12
8	$D_{4h}$	$K_{h}$	Null	$D_{\infty h}$	Null	$D_{4h}$	6	8	7
9	$D_{\infty h}$	$K_{h}$	Null	$D_{\infty h}$	Null	$D_{\infty h}$	5	7	6
10	$T_{h}$	$K_{h}$	Null	Null	$T_{h}$	$O_{h}$	Null	5	4
11	$O_{h}$	$K_{h}$	Null	Null	Null	$O_{h}$	3	3	3
12	$K_{h}$	$K_{h}$	Null	Null	Null	Null	2	2	2
